# Stabilisation of Lutein and Lutein Esters with Polyoxyethylene Sorbitan Monooleate, Medium-Chain Triglyceride Oil and Lecithin

**DOI:** 10.3390/foods10030500

**Published:** 2021-02-26

**Authors:** Zala Gombač, Ilja Gasan Osojnik Črnivec, Mihaela Skrt, Katja Istenič, Andreja Knez Knafelj, Igor Pravst, Nataša Poklar Ulrih

**Affiliations:** 1 Department of Food Science and Technology, Biotechnical Faculty, University of Ljubljana, Jamnikarjeva 101, SI-1000 Ljubljana, Slovenia; zala.gombac@gmail.com (Z.G.); gasan.osojnik@bf.uni-lj.si (I.G.O.Č.); mihaela.skrt@bf.uni-lj.si (M.S.); 2 Valens d.o.o., Poslovna cona A35, SI-4208 Šenčur, Slovenia; katja.istenic@valens.si (K.I.); andreja.knezknafelj@valens.si (A.K.K.); 3 Nutrition Institute, Tržaška Cesta 40, SI-1000 Ljubljana, Slovenia; igor.pravst@nutris.org; 4 Centre of Excellence for Integrated Approaches in Chemistry and Biology of Proteins (CipKeBiP), Jamova 39, SI-1000 Ljubljana, Slovenia

**Keywords:** lutein, lutein esters, encapsulation, emulsions, polyoxyethylene sorbitan monooleate, lecithin

## Abstract

Lutein is a challenging compound to incorporate into food, as it is poorly soluble and unstable in aqueous solutions. In this study, the aim was to prepare stable encapsulates of lutein and lutein esters using feasible and straightforward techniques. Fine suspensions based on polyoxyethylene sorbitan monooleate and medium-chain triglyceride oil micelle-like units with 3.45% lutein esters or 1.9% lutein equivalents provided high encapsulation efficiencies of 79% and 83%, respectively. Lutein encapsulated in fine suspensions showed superior stability, as 86% was retained within the formulation over 250 days at 25 °C in the dark. Under the same storage conditions, only 38% of lutein remained in corresponding formulations. Higher encapsulation efficiencies were achieved with lecithin emulsions, at up to 99.3% for formulations with lutein, and up to 91.4% with lutein esters. In lecithin emulsions that were stored for 250 days, 17% and 80% of lutein and lutein esters, respectively, were retained within the formulations.

## 1. Introduction

**Figure 1:**
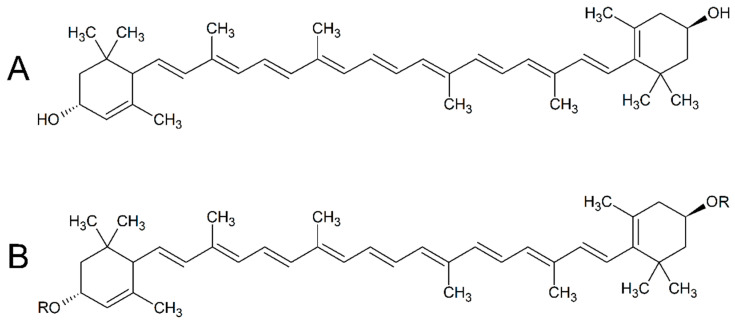
Structural formulae of lutein (**A**) and lutein esters (**B**) (common examples: R = palmitate, myristate, linoleate, laureate).

Lutein (β,ε-carotene-3,3′-diol; C_40_H_56_O_2_; Figure 1A) is a xanthophyll carotenoid that is found in many fruits, vegetables, and flowers. Depending on its tissue concentrations, it provides many yellow, orange, and red colours in plants. This natural pigment is also used as a food colourant (food additive code: E 161b) in a wide array of food products and beverages. As a dietary substance, lutein is important for eye health and skin pigmentation [1,2,3,4,5,6,7,8].

Marigold (*Tagetes erecta*) flowers contain high amounts of carotenoids, which are predominantly fatty esters and diesters of lutein (0.1–0.2% [*w/w*] dry matter as carotenoids, of which 80% are lutein diesters) (Figure 1B) [9]. In the food industry, lutein is usually extracted from marigold flowers either as its fatty acid esters and diesters (e.g., palmitate, myristate linoleate, laureate) or as free lutein when the extraction is combined with saponification ([4,10,11,12,13]. Furthermore, lutein esters are less sensitive to degradation than lutein, with its diesters being more stable than its monoesters [4].

As well as its colouring and colour-enhancing characteristics, lutein has important chemical and biochemical properties that are relevant for the maintenance of eye health.

In this respect, lutein has two important functions: As a filter of high energy blue light, and as an antioxidant and free radical scavenger. It can thus protect the human body from different types of free radicals, with protection of the skin and eyes from photodamage [2], combined with anti-cancer activity and cardiovascular protection [1,2,3,4,5]. The Joint FAO/WHO Expert Committee on Food Additives set the recommended daily intake for lutein at 0 mg/kg to 2 mg/kg body weight [14]. Based on additional scientific data, the acceptable daily intake was subsequently set at 1 mg/kg body weight by the European Food Safety Authority [15]. Furthermore, there are several studies that have reported that daily supplementation of the diet with ~10 mg lutein per adult might provide various health benefits, such as protection from age-related macular degeneration and cataracts [12,16,17,18,19]. However, it should be noted that the strength of the available evidence currently does not substantiate the use of lutein in terms of these nutrition and health claims [20].

As a fat soluble compound, lutein is not soluble in aqueous medium (i.e., solubility in water at 37 °C, 0.197 mg/L; [21]) which is a considerable limitation to its incorporation into commercial food and beverage products. Furthermore, it is susceptible to degradation in the presence of oxygen, light, and/or increased temperatures. Therefore, there is the need for the development of techniques to improve its stability and to enable its incorporation into water-based matrices [11,12,21,22]. To overcome these issues, encapsulation is known to efficiently prevent physicochemical degradation. Delivery systems developed for lutein-based compounds include solid lipid nanoparticles, nanocrystals, nanoliposomes, and various emulsions.

Colloidal delivery systems based on mixtures of oil, water, and surfactants are relevant for compound protection and delivery in food and beverage products, such as microemulsions, nanoemulsions, and conventional emulsions. These can be easily made from food-grade ingredients using relatively simple processes, such as mixing and homogenisation at ambient or elevated temperatures. The oil phase of these colloidal systems can also be used to dissolve lipophilic bioactive compounds, to improve their stability and distribution in aqueous systems [23]. In emulsions, lutein can dissolve in the oil droplets, and this formulation can then be mixed with water. For example, it has been shown that the loss of lutein was higher when entrapped in a single layer emulsion compared to a layer-by-layer emulsion, which was stabilised using gum Arabic [24,25]. Frede et al. [19] compared six types of emulsifiers made with lecithin (i.e., whey protein, β-lactoglobulin, whey protein hydrolysate Biozate 1, and their combinations). The emulsions with combinations of emulsifiers with lecithin gave the best results, due to the small droplet sizes, resistance to creaming, and physical stability. Khalil et al. [10] showed that lutein esters can be protected from thermal degradation by addition of medium-chain triglyceride (MCT) oil, while MCT-oil-based emulsions prevented degradation by UV light. Another study investigated the binding of lutein in milk-protein complexes, and showed that sodium caseinate is a better carrier for lutein than whey protein isolate [26]. Similarly, Davidov-Pardo et al. [17] demonstrated prolonged storage and improved pH and temperature stability of lutein-enriched caseinate emulsions.

Nonionic surfactants, such as poly(oxyethylene) sorbitan esters (i.e., the Tween non-ionic surfactant family) have also been widely used to prepare microemulsions, due to their relatively low cost and efficient emulsifying properties [23]. Polyoxyethylene sorbitan monooleate (PSM; also known as Tween 80) is a widely established aliphatic non-ionic surfactant that is derived from polyethoxylated sorbitan and oleic acid. It consists of hydrocarbon aliphatic residues (i.e., non-polar tails) and polyoxyethylene groups (i.e., polar regions) attached to sorbitan. PSM is approved by the US Food and Drug Administration for parenteral, oral, and topical applications [27], and is included on the European Union list of food additives under E 433 (E 432–436). Zhao et al. [8] prepared lutein-loaded particles with polyvinylpyrrolidone and PSM as the emulsion stabilisers. Their formulation provided good encapsulation efficiency and good water solubility compared to lutein dispersed in water alone, due to the hydrogen bonding between the carrier and the active compound. In a similar approach, zein nanoparticles loaded with 7.5% lutein were stabilised by lecithin and pluronic F127 surfactants. In the presence of these surfactants, the nanoparticles prepared were larger and had a narrower size distribution and stronger zeta potential, and improved encapsulation efficiency [24].

Similarly, other carriers that undergo self-assembly and/or have compatibility with hydrophobic and hydrophilic compounds can be used here. For example, a suitable candidate is lecithin, which is a phospholipid mixture that contains two non-polar hydrocarbon chains and a zwitterionic polar head group that has both positive and negative charges that derive from its amine and phosphate groups. Lecithin is widely applied as an emulsifying agent in cosmetics, food (food additive code E 322), and pharmaceutical products, due to its low toxicity, biocompatibility, and generally recognized-as-safe regulatory status [23].

This short overview demonstrates that encapsulation is a promising route for protection of lutein from degradation during food processing and storage. However, the development of efficient and feasible procedures for such encapsulation of lutein-based compounds is still required, in particular for liquid formulations with lutein, which are rarely available on the market. Thus, the aim of the present study was to prepare stable and water-soluble encapsulates of lutein with commercially available 20% lutein and 20% lutein esters powder extracts. We focused on the development of straightforward and feasible approaches with PSM, MCT oil, and sunflower lecithin as the carrier materials. These were prepared as long-lasting water-miscible formulations that are suitable for incorporation into liquid products, and ultimately to develop formulations that are appropriate for industrial production. The materials produced were characterised in terms of particle size, morphology, encapsulation efficiency, and short-term and long-term stability. Different storage conditions were examined, which included stability tests that were performed over up to 300 days at 25 °C in the dark.

## 2. Materials and Methods

### 2.1. Materials

The following materials were used: Lutein (pharmaceutical secondary standard certified reference material; PHR1699; Sigma Aldrich, St. Louis, MO, USA); powdered marigold (*Tagetes erecta* L.) flower lutein extract (≥20.0% lutein content; henceforth as ‘lutein extract’; Gonmisol, Barcelona, Spain); lutein esters extract (≥20.0% lutein esters content; henceforth as ‘lutein esters extract’; Parry Nutraceuticals, Chennai, Tamil Nadu, India); PSM (59924 [Tween 80]; Sigma Aldrich); MCT oil (Miglyol 812N [F]; IOI Oleochemical Nutrition, Hamburg, Germany); Lipoid H20 (fat-free sunflower lecithin with 20% phosphatidylcholine; Lipoid, Ludwigshafen am Rhein, Germany); xylitol (≥99.0%; X3375; Sigma Aldrich); sodium benzoate (≥99.0%; 71300; Sigma Aldrich); orange concentrate (14.66.001; Presad, Mirna, Slovenia); orange aroma (02 940; Frutarom Etol, Škofja vas, Slovenia); α-tocopherol (vitamin E; ≥87.25%; Shanghai Freemen, Shanghai, China); KOH (Itrij d.o.o., Kropa, Slovenia); NaCl (≥99.5%; 71380; Fluka, Seelze, Germany); acetonitrile (HPLC grade, ≥99.9%; 34851; Honeywell, Seelze, Germany); methanol (HPLC grade, ≥99.8%; 106018; Merck, Darmstadt, Germany); ethanol (absolute; 111727; Merck); ethyl acetate (≥99.5%; 109623; Merck); and n-hexane (≥99.0%; 104367; Merck). Ultrapure water (18.2 MΩ cm, at 25 °C) was used to prepare all of the aqueous solutions, and for all of the other requirements.

### 2.2. Methods

#### 2.2.1. Preparation of the Formulations

Essentially, three types of formulations were prepared as pastes, finely dispersed suspensions, and viscous emulsions (Table 1) to provide protection to the lutein and lutein esters within various food matrices.

Formulation 1LE was prepared as an emulsion paste with the lutein esters extract and PSM. PSM (9 g) was heated to 80 °C, and 2 g lutein esters extract was added, and the mixture was stirred at 800 rpm for an additional 10 min, at 80 °C. The mixture was then cooled in an ice bath.

Formulation 2 was prepared as a finely dispersed suspension based on the principle described by Behnam [28] for obtaining micelle-like units with either the lutein extract (formulation 2L) or the lutein esters extract (formulation 2LE), and PSM and MCT oil. The initial steps were performed as for formulation 1LE. After the 10 min stirring at 80 °C and 800 rpm, 600 mg MCT oil was added to the PSM plus lutein or lutein esters, and the stirring was continued for another 5 min. The mixtures were cooled in an ice bath. Initially, a lutein:MCT oil mass ratio of 1:3 was used (formulation 2L). Additional lutein:MCT oil mass ratios (1:1.5, 1:1.6, 1:3.2) were also investigated (formulations 2L-1, 2L-2, 2L-3, respectively). The initial examined ratios were selected based on our preliminary tests, where an excess of extract or a deficiency of MCT oil (high lutein:MCT oil ratio) resulted in inadequate solubility in water, whereas to high concentrations of MCT oil (low lutein:MCT oil ratio) resulted in its coalescence and layer separation of the formulation.

Formulation 3 was prepared as a viscous emulsion with either the lutein extract (formulation 3L) or the lutein esters extract (formulation 3LE) in a lecithin carrier. Here, a 30 mL solution of ultrapure water, xylitol (15 g) and sodium benzoate (0.095 g) was prepared. A 10-mL aliquot of this solution was then gradually added to lecithin (5 g) while stirring. When the preparation was well mixed, either the lutein extract or the lutein esters extract (1 g) was added, followed by drop-wise addition of the remaining 20 mL of the xylitol and sodium benzoate solution. The mixing was then continued on a stirring plate at 800 rpm for an additional 2 h at 25 °C. In formulation 3L-VE, vitamin E (α-tocopherol; 0.018 g) was also added during the addition of the lutein extract to formulation 3LE, to improve stability.

#### 2.2.2. HPLC

The lutein and hydrolysed lutein esters’ contents were determined using high-performance liquid chromatography (Infinity 1260 system; Agilent Technologies, Santa Clara, CA, USA), with a C18 precolumn (Eclipse XBD-C18; 4.6 × 12.5 mm; ID, 5 μm) and column (Zorbax Eclipse Plus C18; 4.6 × 150 mm; ID, 3.5 μm) (both from Agilent Technologies). The mobile phase was acetonitrile:methanol (90:10; *v/v*), with an injection volume of 10 µL. The elution was isocratic over 15 min at a flow rate of 0.8 mL/min. The analysis was performed at 30 °C (sample temperature, 20 °C) and the eluted components were determined with a UV-Vis diode array detector, at 446 nm. Lutein was identified and quantified according to retention time and absorption spectra, compared to the lutein standard. All of the analyses were performed in triplicate. The procedures for the calibration curves and sample preparation are described in the Supplementary Materials.

#### 2.2.3. Encapsulation Efficiency

The encapsulation efficiencies were calculated from the total (*m*_1_) and free (*m*_2_) lutein contents, as described in Equation (1). Free lutein was determined by dissolving either 250 mg (formulations 1LE, 2L, 2LE) or 1 mL (formulations 3L, 3LE) samples in 10 mL ultrapure water. An aliquot of 1 mL was then mixed by vortexing and centrifuged at 18,500× *g* for 1 h. The supernatant was transferred into absolute ethanol (formulations 1LE, 2L, 2LE), ethyl acetate (formulation 3L) or hexane (formulation 3LE), and diluted with the mobile phase for HPLC. Total lutein was determined by the procedures described in the Supplementary Materials (‘Preparation of the samples with lutein’).

(1) $$Encapsulation\;efficiency\;\left[\%\right]=\frac{m_{1}-m_{2}}{m_{1}}\times 100$$

#### 2.2.4. Stability Tests

Stability tests were performed for all the formulations prepared, as well as for the free lutein and free lutein esters. The stability of the free lutein and free lutein esters was examined in the dark for 7 days at 25 °C, and for 76 days at 4 °C. As the formulations were designed to withstand more rigorous conditions, the protection of the lutein and lutein esters in formulations was further examined for prolonged storage times under ambient conditions. Initially, the stability tests were performed simultaneously with all of the formulations for 60 days at 25 °C in the dark. Long-term stability tests were then performed and concluded when at least ~20% degradation of the encapsulated compound was observed (at ~300 days).

In the same manner, three additional stability tests were performed with the lecithin emulsions (formulation 3):

1. Storage comparison: At 4 °C in the dark versus at 25 °C under a light source placed 20 cm above the sample (fluorescent light: 20 W, 6400 K, 220–240 V, 50/60 Hz; with pronounced spectral intensities at 620, 550, 440, 405 nm, in order of relative power).
2. Vitamin E: Preparation of the lutein extract emulsion without (3L) and with (3L-VE) vitamin E, and comparison of storage at 25 °C in the dark.
3. Accelerated storage: Simulation of accelerated conditions of storage (Incucell LSIK-B2V/IC 55 storage chamber; MMM Medcenter Einrichtungen GmbH, Germany). In the storage chamber, 1 day at 37 °C corresponded to ~4 days of storage in real time at 25 °C.

During all of these tests, the samples were placed in sealed inert containers, and all of the experiments were performed in triplicate.

#### 2.2.5. Colour Measurement

The changes in the colour of the lutein esters formulations were measured at 25 °C with an instrumental colorimeter (Chroma meter CR-400; Konica Minolta, Japan). The parameters measured identified the differences in lightness and darkness (parameter L*), red and green (parameter a*), and yellow and blue (parameter b*). From these, the differences in the colour (ΔE) and the differences in the colour intensities (ΔC*) were calculated as cumulative values. For the analysis, 100 mg of each sample was spread between two microscope slides and the colour parameters were recorded against a white background. Further description of the parameters and calculations is provided in the Supplementary Materials.

#### 2.2.6. Particle Diameter and ζ-Potential

The physical stability of the formulations was quantified by measuring the changes in the mean droplet diameter and ζ-potential during storage. These measurements were performed using an analyser (Zetasizer Nano ZS; Malvern Instruments, Malvern, UK). For these measurements, 10 mg samples were diluted in 10 mL ultrapure water. The diffusion barrier method was used, where 20 µL of each sample was introduced into the bottom of the folded capillary cell, which was pre-filled with water. All of the measurements were carried out in triplicate, at 25 °C.

#### 2.2.7. Laser Confocal Scanning Microscopy

Three-dimensional (3D) microscopy was performed with a laser microscope (Lext OLS5000; Olympus). Here, 10 mg formulations 1LE or 2LE were diluted in 10 mL ultrapure water, and then thinly and gently spread on an observation slide with a glass cylinder. Viscous emulsions (i.e., 3LE) were not observed, as they could not be subjected to drying. After drying, the particle diameters were estimated as the mean size measurements across at least 50 different particles in the viewfinder, chosen at random.

## 3. Results and Discussion

### 3.1. Encapsulation Efficiency

Encapsulation efficiency was determined for the eight different formulations of the lutein and lutein esters with PSM and MCT oil or lecithin (Table 2). The composition of the delivery system markedly affected its properties. Overall, all of these encapsulation systems provided high encapsulation efficiencies, in the order of pastes < fine suspensions < emulsions. For the viscous emulsions, nearly complete encapsulation of the lutein was achieved (formulation 3L), with >90% of the lutein esters also entrapped (formulation 3LE).

In the pastes and suspension formulations for the lutein esters (1LE and 2LE, respectively), the encapsulation efficiencies were similar, at just under 80%. For lutein, various finely dispersed suspensions (2L, 2L-1 to 2L-3) were prepared. Decreasing the proportion of PSM (to MCT) from formulations 2L to 2L-1 resulted in 10% reduction in encapsulation efficiency. Conversely, increasing the proportion of MCT oil (to PSM) as formulations 2L vs. 2LE, and 2L-2 vs. 2L-3) had a minor influence on the encapsulation efficiency. This might indicate that low proportions of MCT oil (formulation 2L-3) were already sufficient in the formation of micelle-like units, and therefore even lower amounts should be further investigated. By changing these proportions of PSM and MCT oil, almost 90% entrapment of lutein was achieved (formulation 2L-2). Moreover, by increasing the lutein concentration from 20% to 50% in the extract, the encapsulation efficiency only increased by 6% (comparing sample formulations equivalent to 2L, data not shown). This relatively small increase in lutein entrapment at its considerably higher input concentration suggests that in formulation 2L, the finely dispersed suspension had already approached its loading capacity, which in turn determined the encapsulation efficiencies obtained.

It should be noted that other studies have determined encapsulation efficiencies for different systems of 87.5% to 93.8% based on concentrated (90%) lutein (e.g., spray-dried water–oil–water emulsions, lutein-loaded polyvinylpyrrolidone particles) [7,8]. Furthermore, Li et al. [29] recently reported an emulsion encapsulation approach where 84.0% encapsulation efficiency was obtained using 90% lutein. Thus, we have demonstrated here that similar encapsulation efficiencies can be achieved with more commercially attractive lutein extracts compared to less feasible pure lutein or highly concentrated lutein powders.

### 3.2. Stability Tests

Based on the observed encapsulation efficiencies, the lutein ester plus PSM paste (formulation 1LE), and the finely dispersed suspensions of lutein (formulations 2L) and lutein esters (formulation 2LE) plus PSM and MCT oil were further tested in stability tests, in parallel to free lutein and lutein esters extracts. These stability tests were performed to gain more information on the potential shelf-life of the products. The non-encapsulated compounds were studied at 4 °C in the dark, as the initial investigation of stability at 25 °C, and these showed rapid degradation over the first 7 days. The goal of encapsulation was to provide formulations that do not require the use of specific storage approaches, such as refrigeration.

Table 3 shows that all of the formulations that contained the lutein and lutein ester extracts were more stable than the non-encapsulated extracts. The best stability was achieved for formulation 3LE. For both the lutein and lutein esters extracts, the addition of lecithin provided more stable systems for the encapsulation. Furthermore, there was up to 40% increase in the loading capacity of the initial active components achieved with lecithin, compared to PSM without or with MCT oil.

### 3.3. Stability of the Lutein and Lutein Esters Extracts and Their Pastes and Fine Suspensions

**Figure 2:**
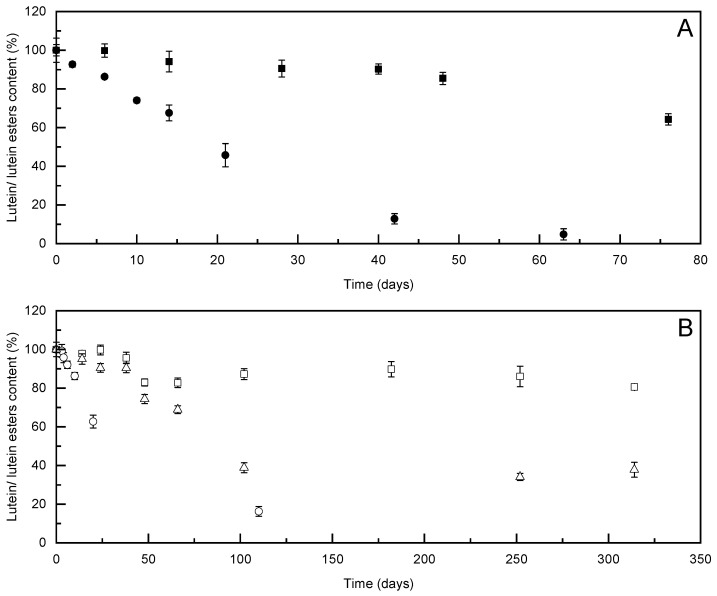
(**A**) Stabilities of lutein extract (●) and lutein esters extract (■) when stored in sealed dark containers at 4 °C. (**B**) Stabilities of formulation 2L with lutein (fine suspension, ○) and formulations 1LE (paste, △) and 2LE (fine suspension, □) with lutein esters when stored in sealed dark containers at 25 °C. Data are means ± SD (*n* = 3).

The prolonged stability for the non-encapsulated lutein and lutein esters extracts was monitored over 80 days at 25 °C (Figure 2A). During this time, the lutein content in the non-encapsulated lutein extract was completely degraded, and the more stable non-encapsulated lutein esters extract reached 35% degradation. These data demonstrated the requirement for encapsulation of these compounds.

### 3.4. Stability of Lutein and Lutein Esters Syrups with Lecithin

Formulations 3 were prepared as viscous emulsions (i.e., in the form of a syrup) with higher concentrations of the active components than in formulations 1 and 2. In the final product developed that was intended for the market, additional ingredients were also added, such as:

(i) Red orange concentrate (8%, *w/w*), apple concentrate (5%, *w/w*) and orange aroma (1.5%, *w/w*): To improve the organoleptic properties, due to the unpleasant taste of the extracts.

(ii) Vitamin E (0.035%, *w/w*): To prolong the stability of the active compounds.

**Figure 3:**
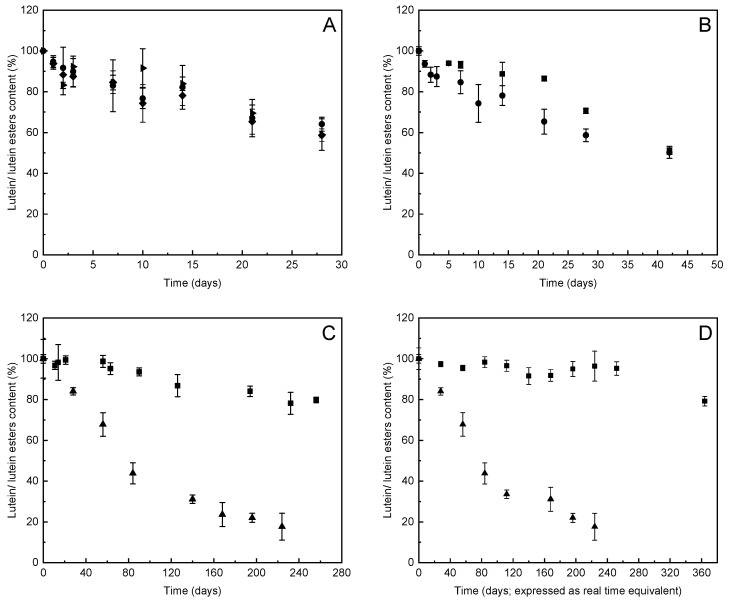
(**A**) Stabilities of formulation 3L (viscous emulsion) with lutein extract over 30 days under different storage conditions: In the light at 25 °C (►); in the dark at 25 °C (♦); and in the dark at 4 °C (●). (**B**) Stabilities of formulation 3L (viscous emulsion) with lutein over 42 days at 25 °C in the dark without (●) and with (■) vitamin E. (**C**,**D**) Long-term stabilities of formulations 3L with lutein (▲) and 3LE with lutein esters (■) following real time at 25 °C in the dark (**C**) and accelerated conditions of storage at 37 °C in the dark (**D**). Time scale is calculated to real time. Data are means ± SD (*n* = 3).

The stabilities of the encapsulated compounds in formulation 3L were monitored for 30 days under three different storage conditions: Exposure to light at 25 °C; at 25 °C in the dark; and at 4 °C in the dark (Figure 3A). The stability of formulation 3L with the addition of vitamin E (formulation 3L-VE) was monitored over 42 days, with these data compared with the formulation without vitamin E (Figure 3B, 3L). In contrast to rapid degradation of the free lutein at 25 °C (data not shown) and at 4 °C (Table 3, Figure 2A), formulation 3L maintained more than 60% of the initial concentration of lutein after 1 month of storage. Relatively comparable protective effects of formulation 3L were observed as storage in the presence of light versus dark, as well as at ambient temperature and with refrigerated storage (Figure 3A).

However, the marked increase in the stability of the active component was still not considered as sufficient for commercial application to storage prolongation. Therefore, to further improve product stability, formulation 3L was reinforced by the addition of vitamin E. Amongst the tested concentrations, the addition of 0.035% (*w/w*) vitamin E provided the longest (10%) improvement to the stability (see Supplementary Materials, Figure S2). However, this formulation contained 50% of initial concentration of lutein after 42 days of storage in the dark at 25 °C (Figure 3B). It appeared that the added vitamin E improved the stability of the lutein in the first 20 days of storage, but it did not improve the long-term stability. This appears to be due to gradual degradation by radical-mediated oxidation of vitamin E, which can occur over the timeframe of days or weeks, depending on the environment conditions [30].

As the formulations with lutein extract without and with vitamin E (3L, 3L-VE, respectively) were not sufficiently stable, formulation 3 was prepared with the lutein esters extract and lecithin (formulation 3LE). Moreover, based on our preliminary sensory evaluations, the lutein esters extract had a stronger, bitter taste in comparison to the lutein extract, and might thus be rejected by consumers. Therefore, to improve the taste, the final formulation 3LE also contained commercially available orange aroma, apple juice, and orange juice concentrates, with the equivalent lutein content to formulation 3L. After 250 days, this improved formulation 3LE maintained 80% of the initial concentration of the lutein esters (Figure 3C). It should also be noted that the same amount of lutein esters in terms of g/L content corresponds to the halved content of lutein after hydrolysis, and furthermore, that esterification of lutein does not impair its bioavailability [31,32]. The final concentrations of lutein in products with lutein esters in our formulation was still sufficient for use in the production of food supplements.

### 3.5. Stability of Lutein and Lutein Esters in Syrups with Lecithin under Accelerated Conditions of Storage

To obtain relevant aging data, and to compare various aging procedures, the stabilities of formulations 3L and 3LE were studied under conditions of accelerated ageing (i.e., at 37 °C), and in real-time (i.e., at 25 °C). Formulations 3L and 3LE were stored under accelerated conditions for at least 56 days (corresponding to ~224 days ageing in real time). After 14 days of accelerated storage (i.e., ~56 days real-time equivalent), the concentration of lutein in formulation 3L started to decrease, and after 56 days of accelerated storage (i.e., ~340 days real-time equivalent), only 17% of the initial concentration remained. The stability of formulation 3L was therefore greatly improved compared to free lutein; however, this was still not sufficient for commercial use. Finally, formulation 3LE with lutein esters provided superior long-term stability, as 95% and 79% of the active components were retained under the conditions of accelerated ageing after 63 days (i.e., ~252 days real-time equivalent) or 91 days (~364 days in real-time), respectively (Figure 3D).

At this point, it is important to note that various intermediates have been reported to form during the degradation of lutein, such as 1,1,6-trimethyl-1,2-dihydronaphtalene, 2,3-dehydro-α-ionone (two isomers), 3,4-dehydro-β-ionone, 3-oxo-α-ionone, and 3-hydroxy-β-ionone [33,34]. However, here, it was not possible to identify any additional compounds by HPLC analysis. The chromatograms of these formulations from the start and during the prolonged storage could only be distinguished by the decreases in areas of the analytes at fixed retention times. Therefore, none of the tested delivery systems saw new peaks on HPLC analysis that would indicate the formation of new compounds (see Supplementary Materials, Figure S3).

### 3.6. Colour Stability

Colour stability measurements were performed during the storage. These colour measurements were performed on the day of preparation, and after 1 week, 3 weeks, and 300 days. These data are shown in Table 4 and Table 5. Only the pastes and the fine suspensions with the lutein esters were tested for colour stability, as colour changes for the syrups are of less relevance here.

Table 4 and Table 5 show that both of the formulations examined here (1LE, 2LE) showed stable colour properties over the 300 days of observation. For formulation 1LE (Table 4), the cumulative ΔE of 2.0 to 2.6 indicates that the colour differences will be perceptible by the human eye through close observation of the sample. For formulation 2LE (Table 5), the small differences in the cumulative parameter, as ΔE <1, means that the colour differences are not perceptible to the human eye. Visual representation of the samples is provided in the Supplementary Materials (Figure S4).

### 3.7. Physical Stability—Particle Size and ζ-Potential

To evaluate the physical stability and suitability of the incorporation of these formulations into food products, the particle sizes and ζ-potentials were determined. The physical stabilities of formulations 1LE, 2LE, and 3LE (all of which contained the lutein esters extract) were monitored by measuring the mean droplet diameter and ζ-potential over 2 months of storage at 25 °C in the dark. These data are shown in Table 6.

Minor changes in particle size and ζ-potential were seen over 50 days of storage at room temperature (Table 6), which indicate good physical stability of the samples, especially for formulations 1LE and 3LE. The mean initial particle sizes were from 800 nm to 850 nm for formulations 1LE and 2LE, and ~400 nm for formulation 3LE. After 50 days of storage, there was ~25% loss in particle size for the fine suspension of formulation 2LE, with only ~5% loss for the paste and the emulsion of formulations 1LE and 3LE, respectively.

The particle sizes of the encapsulation formulations depended on the preparation technique, wall materials, and compound–carrier interactions. In the literature, similar approaches that have been applied to lutein encapsulation have included coconut skim milk and whey-protein-stabilised oil–water emulsions [10,35] and lipid-core nanocapsules [11], which reached particle diameters of 0.4 µm to 0.5 µm, 0.7 µm to 1.0 µm, and 0.2 µm, respectively. In the same way, our approaches here produced small particle sizes with good delivery properties, and demonstrated the production of relatively developed morphologies via this straightforward preparation of the paste, fine suspensions, and emulsions.

The ζ-potential with PSM was measured at the beginning and end of the storage periods here, for each the samples taken through the test period. The ζ-potential was similar for the formulations with (formulation 2LE, fine suspension) and without (formulation 1, paste) MCT oil, at −10 mV to −15 mV, and was even weaker for the emulsions (formulation 3LE; around −1 mV). Similarly, the ζ-potential for the lutein-loaded nanocapsules of Brum et al. [11] were reported to have correspondingly low values (i.e., −5.1 ± 2.22 mV). The particle sizes and ζ-potentials measured here indicated that the particles would remain stable within the suspension, where they would sediment out, which can be prevented by increasing the particle charge, if required. Li et al. [29] investigated lutein-enriched emulsions that they stabilised with sodium caseinate using a high-pressure homogenisation process, for the influence of the environment conditions on the particle size. Their optimised lutein nanoemulsion droplet mean diameter was 234 ± 3.4 nm, and their ζ-potential was −36.6 ± 1.5 mV, where decreased particle size and increased ζ-potential were achieved by modulation of the homogenisation process, as well as the pH and ionic strength, which demonstrated a similar approach to the present study, where such changes might also be suitable for our delivery systems.

### 3.8. Microscopy

As a complementary method to dynamic light scattering, dynamic laser 3D microscopy was performed to evaluate the morphology of the formulations and the particle sizes. This showed that the particle sizes for formulations 1LE and 2LE were 2.3 ± 0.9 µm and 1.9 ± 0.9 µm, respectively (see Supplementary Materials, Figure S6). In comparison, the particle sizes measured by dynamic light scattering were approximately half these values for both of these formulations. This is consistent with our own [36] and other studies [37,38,39,40], where these previous observations have indicated that different particle sizes can be measured using different methods. For example, hydration has an important role in particle size determinations. When measured in solution, particles are often smaller, as a semi-fluid layer can form over the outer region of the particle. For this reason, it is important to check the particle size by at least two methods, to gain relevant insight into the particle morphology. Furthermore, 3D microscopy shows that emulsions included bimodal distributions of smaller (~0.1–0.6 µm) and larger (~2–8 µm) particles.

## 4. Conclusions

In the present study, we have developed an inexpensive, straightforward, and rapid method for encapsulation of extracts of lutein and lutein esters. For the lutein extracts, all of the formulations provided protection of this active compound, and are suitable for products with shelf-lives <1 month. Lutein esters are known for their superior stabilities compared to lutein. Our data here show that the lutein esters are also likely to be better incorporated into these carriers, as indicated by the encapsulation efficiencies (80–90%), the various stability tests, and the evaluations of solubility. For the lutein esters, the paste with PSM (formulation 1LE) provided medium protection from degradation, enabling storage periods approximately 4-fold longer than for the pure compounds. Moreover, both the PSM and MCT oil finely dispersed suspensions (formulation 2LE) and the lecithin emulsion (formulation 3LE) provided the most stable formulations with the lutein esters (300 days storage, up to 80% remaining). The pastes do not appear to greatly prolong the stability of the lutein esters, but did provide a basic means of protection. All of these formulations were physically stable, as the particle sizes and colour parameters were maintained over several months at room temperature in the dark. Due to the bitter taste of the lutein esters, there is the need for the addition of masking agents and flavours in the final products. The paste and finely dispersed suspensions show potential for use in products such as fruit drinks, tea, and other beverages. The emulsions prepared here can be used in a wide variety of food products. Among all of these formulations, the lutein esters in the micellar suspensions and emulsions showed the best overall properties and are the most promising for industrial production and applications, as liquid lutein products remain scarce on the market. In our further ongoing studies, we will focus on the lutein esters in emulsions in terms of prolonged long-term stability studies and planned clinical testing.

## 5. Patents

Patent pending for lutein equivalent delivery systems from different sources and the protocol for their production (Slovenian national patent application P-202000229, submitted on 8 December 2020).

## Figures and Tables

**Table 1:** Formulations used to protect the lutein (-L suffix) and lutein esters (-LE suffix) in the various food matrices.

Formulation	Extract	Carrier	Form
1LE	Lutein ester	Polyoxyethylene sorbitan monooleate	Paste
2L, 2L-1/2/3	Lutein	Polyoxyethylene sorbitan monooleate, medium-chain triglyceride oil	Finely dispersed suspensions
2LE	Lutein ester	Polyoxyethylene sorbitan monooleate, medium-chain triglyceride oil	Finely dispersed suspensions
3L	Lutein	Lecithin	Viscous emulsion
3L-VE	Lutein	Lecithin, vitamin E	Viscous emulsion
3LE	Lutein ester	Lecithin	Viscous emulsion

**Table 2:** Encapsulation efficiencies of the formulations with lutein/lutein esters.

Condition/Formulation	Carrier	Defined Lutein/Lutein Esters:MCT Oil Mass Ratio	Calculated Lutein/Lutein Esters Content (%)	Calculated Lutein:Carrier Mass Ratio	Measured Encapsulation Efficiency (%)
Paste/					
1LE	PSM	/	3.45	1:22.5	77.8 ± 0.19
Fine suspension/					
2L	PSM	1:3	3.28	1:22.5	83.1 ± 0.26
2L-1	PSM	1:1.5	2.27	1:37.5	73.5 ± 0.16
2L-2	PSM	1:1.6	3.65	1:23.8	89.9 ± 0.37
2L-3	PSM	1:3.2	3.45	1:23.8	89.2 ± 0.22
2LE	PSM	1:1.5	3.45	1:22.5	79.8 ± 0.19
Viscous emulsion/					
3L	Lecithin	/	1.9	1:28.3	99.3 ± 0.06
3LE	Lecithin	/	1.9	1:28.3	91.4 ± 0.01

Data are means ± SD (*n* = 3). For formulation details, see Table 1. PSM, polyoxyethylene sorbitan monooleate; MCT oil, medium-chain triglyceride oil.

**Table 3:** Stabilities of the formulations with lutein/lutein esters during storage at 25 °C in the dark.

Condition/Formulation	Sample	Stabilities over Time (%)			
		0 Days	20 Days	40 Days	60 Days
Free extract/					
-	Lutein	100	45.7 ± 0.6	12.8 ± 0.3	4.8 ± 0.3
-	Lutein esters	100	90.5 ± 0.4	85.4 ± 0.3	64.2 ± 0.3
Paste/					
1LE	Lutein esters	100	90.5 ± 0.2	74.3 ± 0.3	68.9 ± 0.2
Fine suspension/					
2L	Lutein	100	62.7 ± 0.3	32.3 ± 0.6	20.3 ± 0.6
2LE	Lutein esters	100	97.8 ± 0.5	82.9 ± 0.2	82.8 ± 0.4
Viscous emulsion/					
3L	Lutein	100	84.0 ± 0.2	67.8 ± 0.6	43.8 ± 0.5
3LE	Lutein esters	100	96.7 ± 0.3	95.2 ± 0.2	93.6 ± 0.5

Data are means ± standard deviation (*n* = 3).

**Table 4:** Colour stability parameters (L*, a*, b*), cumulative difference in colour (ΔE), and cumulative difference in colour intensity (ΔC*) of lutein esters in the PSM paste (formulation 1LE).

Parameter	Stabilities over Time (%)			
	0 Days	7 Days	21 Days	300 Days
L*	47.3 ± 0.2	48.2 ± 0.2	45.8 ± 0.8	48.5 ± 0.3
a*	30.4 ± 0.5	30.8 ± 0.3	30.2 ± 0.6	30.2 ± 0.2
b*	42.0 ± 0.6	44.1 ± 0.4	39.3 ± 1.0	44.4 ± 0.2
ΔE	0	2.3	3.1	2.6
ΔC*	0	2.0	2.7	2.4

Data are means ± standard deviation (*n* = 3).

**Table 5:** Colour stability parameters (L*, a*, and b*), cumulative difference in colour (ΔE), and cumulative difference in colour intensity (ΔC*) of lutein esters in the PSM and MCT oil fine suspensions (formulation 2LE).

Parameter	Stabilities over Time (%)			
	0 Days	7 Days	21 Days	300 Days
L*	46.2 ± 0.2	46.3 ± 0.5	48.1 ± 1.0	45.8 ± 0.4
a*	33.6 ± 0.1	34.3 ± 0.5	25.6 ± 0.4	33.7 ± 0.14
b*	42.7 ± 0.6	42.2 ± 0.6	43.9 ± 1.0	42.5 ± 0.5
ΔE	0	0.9	8.3	0.5
ΔC*	0	0.9	8.1	0.3

Data are means ± standard deviation (*n* = 3).

**Table 6:** Particle sizes and ζ-potentials during storage for the formulations of lutein esters with PSM, PSM, and MCT oil, or lecithin (formulations 1LE, 2LE, and 3LE, respectively).

Condition/Formulation	Parameter	Time (Days)			
		0	10	28	50
Paste/					
1LE	Size (d. nm)	848 ± 133	882 ± 200	911 ± 197	882 ± 205
	ζ-Potential (mV)	−12.4 ± 1.7	-	-	−11.4 ± 3.8
Fine suspension/					
2LE	Size (d. nm)	817 ± 195	722 ± 160	907 ± 267	618 ± 17
	ζ-Potential (mV)	−16.5 ± 3.3	-	-	−15.0 ± 4.1
Viscous emulsion/					
3LE	Size (d. nm)	380 ± 71	375 ± 65	370 ± 69	365 ± 56
	ζ-Potential (mV)	−1.5 ± 4.6	-	-	−1.1 ± 3.6

Data are means ± standard deviation (*n* = 3).

## Data Availability

The data presented in this study are available on request from the corresponding author.

## Supplementary Materials

The following are available online at https://www.mdpi.com/2304-8158/10/3/500/s1, Figure S1: Stability of the lutein esters in the extract (A) and in ethanol solution at 1 mg/mL (B) under the different storage conditions (as indicated). Data are means ± standard deviation (*n* = 3); Figure S2: Stability of lutein in formulation 3L with various additions of vitamin E (as indicated), with storage at room temperature and in the dark. Data are means ± standard deviation (*n* = 3); Figure S3: Representative HPLC chromatograms of the lutein standard in solution and the lutein esters formulations before and after storage (as indicated). The lutein esters were hydrolysed before the analysis, and were thus detected as free lutein. The peaks at 6.3 min corresponded to lutein, with no further peaks detected for the lutein standard. The peak at 8.5 min only appeared when the hydrolysed lutein esters were analysed; Figure S4: Colours of the lutein esters in the paste (formulation 1LE) and the fine suspension (formulation 2LE) during storage; Figure S5: Stabilities of 20 mg/L lutein esters in formulations 1LE (▲) and 2LE (■) in water when stored at room temperature in the dark. Data are means ± standard deviation (*n* = 3). The lutein esters concentrations were determined from absorbance at 446 nm. The lutein esters were more stable in formulation 2LE than in formulation 1LE. The water dispersion of formulation 2LE contained 85% of the initial lutein esters concentration after 80 days, while that of formulation 1LE contained only 65% after the same time period. Formulation 2LE was expected to be more stable in water than formulation 1LE, as it was already more stable in the prepared form. It appears that addition of the medium-chain triglyceride oil provided greater protection due to the entrapment within micelle-like units; Figure S6: Micrographs of samples 1LE (A and C) and 2LE (B and D) via light microscopy at 228 × magnification (A and B) and corresponding 3D laser scans at 452 × magnification (C and D).
