# Supplementary material for: Stabilisation of Lutein and Lutein Esters with Polyoxyethylene Sorbitan Monooleate, Medium-Chain Triglyceride Oil and Lecithin
Source: Foods. 2021 Feb 26;10(3):500. doi: 10.3390/foods10030500 (PMC7996776; doi:10.3390/foods10030500)
Supplement: Supplementary file 1 [file foods-10-00500-s001.pdf]

Stabilisation of lutein and lutein esters with polyoxyethylene sorbitan monooleate, medium-chain triglyceride oil and lecithin

Supplementary Materials

Zala GOMBAČ [a-], Ilja Gasan OSOJNIK ČRNIVEC [a], Mihaela SKRT [a], Katja ISTENIČ [b], Andreja KNEZ KNAFELJ [b], Igor PRAVST [c], Nataša POKLAR ULRIH [ad*]

[a] Chair of Biochemistry and Food Chemistry, Department of Food Science and Technology, Biotechnical Faculty, University of Ljubljana, Jamnikarjeva 101, SI-1000 Ljubljana, Slovenia

[b] Valens, d.o.o, Poslovna cona A35, SI-4208 Šenčur, Slovenia

[c] Nutrition Institute, Tržaška cesta 40, SI-1000 Ljubljana, Slovenia

[d] Centre of Excellence for Integrated Approaches in Chemistry and Biology of Proteins (CipKeBiP), Jamova 39, SI-1000, Ljubljana, Slovenia

[-] Affiliation at the time of the study.

*Corresponding author (natasa.poklar@bf.uni-lj.si)

1. HPLC

1.1 Preparation of calibration curves

The calibration curves were prepared by dissolving the pharmaceutical standard of lutein in absolute ethanol for the stock solution at 400 µg/mL. External standard concentrations of 0.8 µg/mL to 200 µg/mL lutein were prepared by diluting the stock solution in the HPLC mobile phase. The stock solution and standard solutions were freshly prepared before each analysis. The lutein contents in the HPLC samples were calculated from the calibration curve of y = 186.23x (R^2 = 0.9954).

1.2 Preparation of samples with lutein

Lutein powder extract (10 mg) was dissolved in 10 mL absolute ethanol and then diluted in the mobile phase at the required ratios. An aliquot of formulation 2L (30 mg) was dissolved in 20 mL absolute ethanol and diluted in the mobile phase. Ethyl acetate (4 mL) was added to 1 mL formulation 3L, vortexed for 30 s, and then centrifuged at 2590× *g* for 10 min. The supernatant was diluted with mobile phase and analysed by HPLC.

1.3 Preparation of samples with lutein esters, and their hydrolysis

Prior to analysis, the lutein esters were hydrolysed to lutein. The lutein ester extracts (10 mg) and 2 mL 0.5 M KOH in ethanol was added to capped test-tubes, and the samples were heated to 60 °C for 1 h. After this hydrolysis, the samples were cooled to room temperature, and then 3 mL ethyl acetate and 1 mL 10% (w/v) NaCl solution were added. Next, the samples were vortexed for 30 s. After phase separation, the upper phase was collected and diluted with HPLC mobile phase. After this procedure, the lutein esters were determined in terms of the free lutein content.

To determine the lutein esters content in samples of the various formulations, 25 mg formulations 1LE or 2LE and 2 mL 0.5 M KOH in ethanol were added to capped test-tubes, and the above mentioned procedure for the hydrolysis of the lutein esters was performed. The lutein esters emulsion with lecithin for formulation 3LE (1 mL) was extracted with 5 mL hexane, three times in succession. After vortexing and centrifugation at 2590× *g* for 10 min, the supernatant was collected in a round-bottomed flask and dried on a rotary evaporator. Then, the above procedure for hydrolysis of the lutein esters was performed.

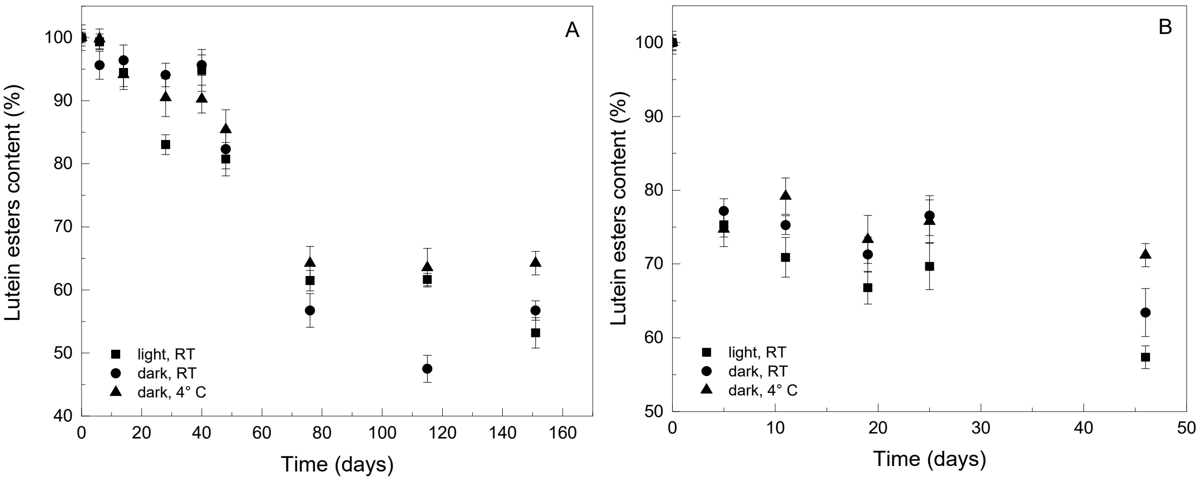

Figure S1: Stability of the lutein esters in the extract (**A**) and in ethanol solution at 1 mg/mL (**B**) under the different storage conditions (as indicated). Data are means ±standard deviation (n = 3).

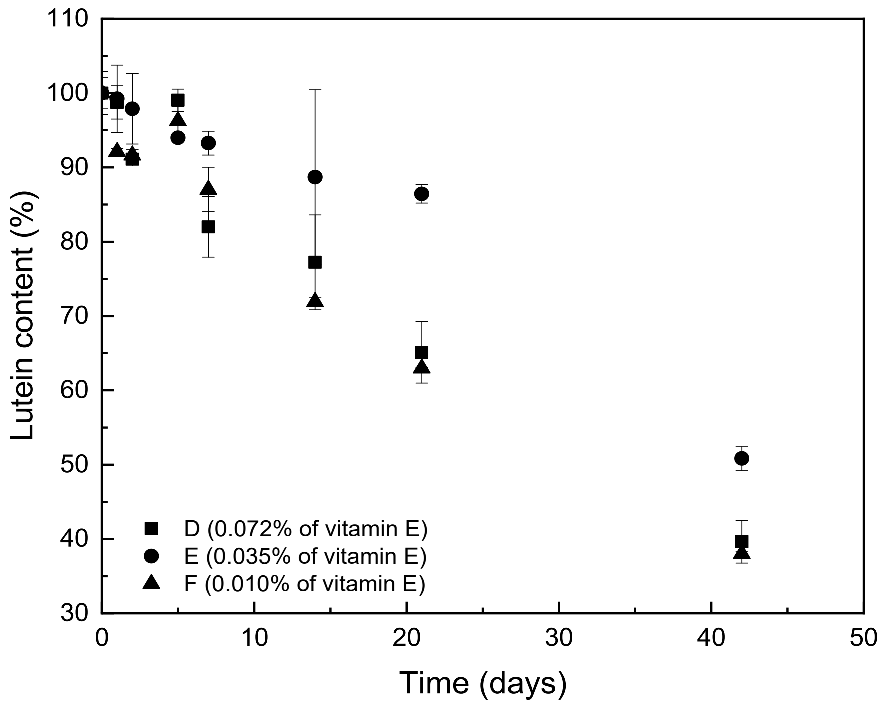

Figure S2: Stability of lutein in formulation 3L with various additions of vitamin E (as indicated), with storage at room temperature and in the dark. Data are means ±standard deviation (n = 3).

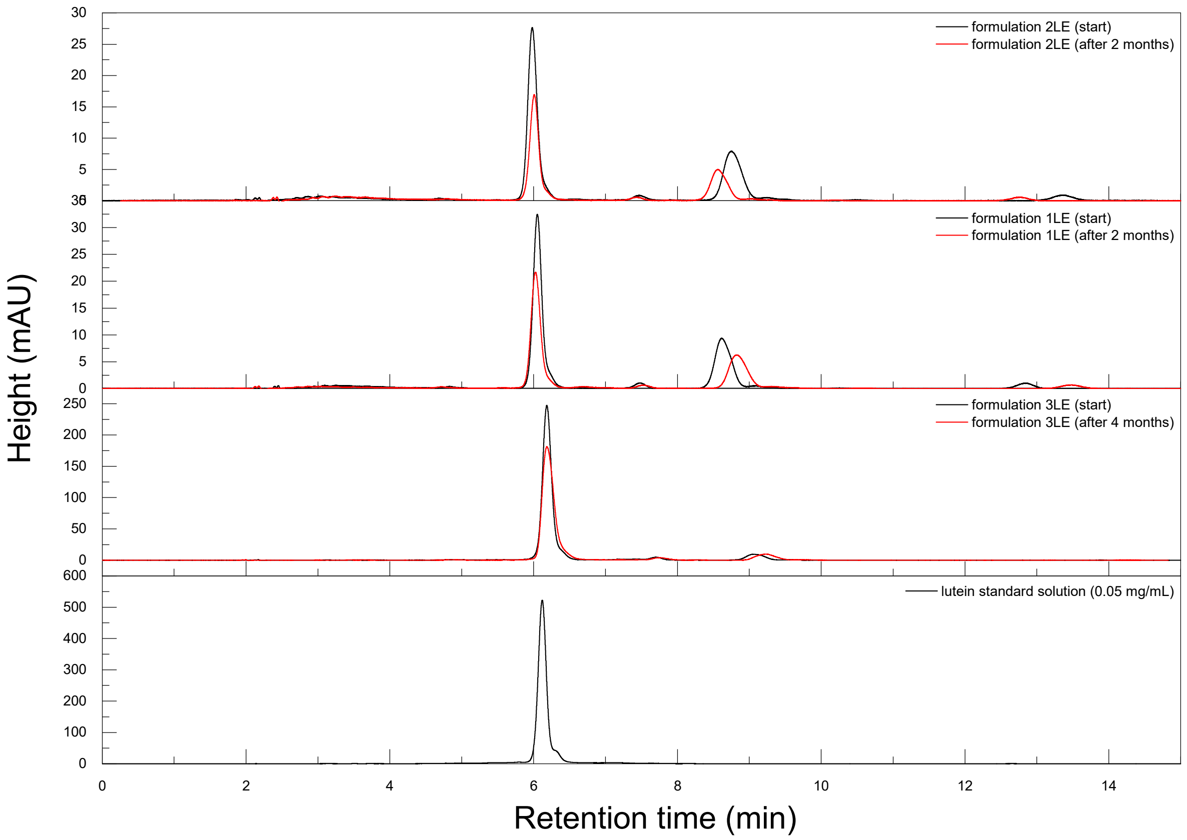

Figure S3: Representative HPLC chromatograms of the lutein standard in solution and the lutein esters formulations before and after storage (as indicated). The lutein esters were hydrolysed before the analysis, and were thus detected as free lutein. The peaks at 6.3 min corresponded to lutein, with no further peaks detected for the lutein standard. The peak at 8.5 min only appeared when the hydrolysed lutein esters were analysed.

2 Colour stability

The colorimeter measured the colour coordinates of the suspensions according to the International Commission on Illumination (CIE) tristimulus system (i.e., L*, a*, b*). L* represents the lightness, and a* and b* represent the colour. L* varies from black = 0 to white = 100, a* from red (a >0) to green (negative <0), and b* from yellow (b >0) to blue (b <0). The total colour difference (ΔE) was calculated from the tristimulus colour coordinates using Equation (S1):

$$\Delta E = \sqrt[2]{(L2^{*} - L1^{*})^2 + (a2^{*} - a1^{*})^2 + (b2^{*} - b1^{*})^2} \quad \text{(S1),}$$

where L_1*, a_1* and b_1* are the initial values of the L*, a* and b* colour coordinates, as measured immediately after sample preparation, and L_2*, b_2* and a_2* are measured after the specific storage period.

Additionally, the differences in the chroma (ΔC*) values, which represents the colour intensities of the sample, were calculated using Equation (S2):

$$\Delta C^{*} = \sqrt[2]{(a2^{*} - a1^{*})^2 + (b2^{*} - b1^{*})^2} \quad \text{(S2).}$$

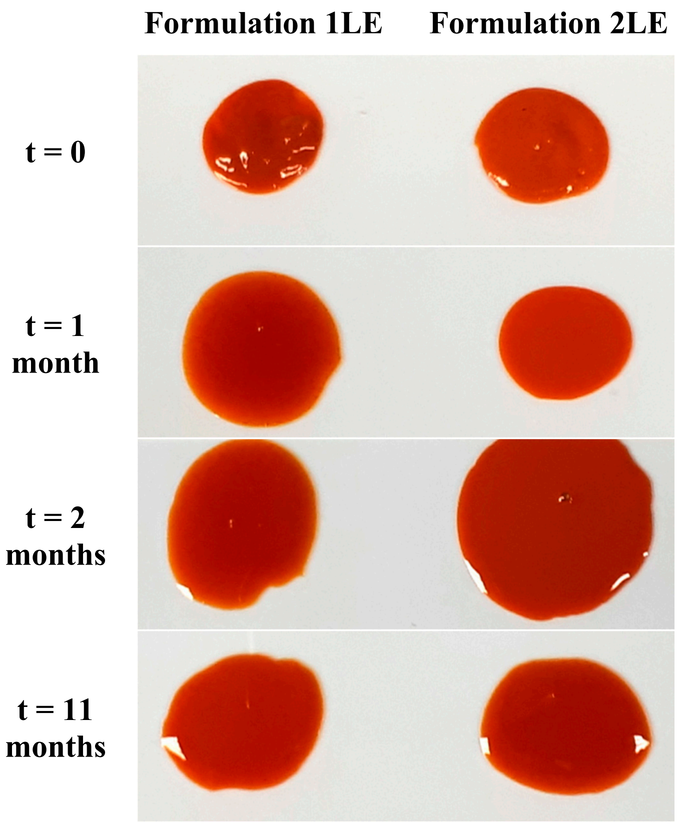

Figure S4: Colours of the lutein esters in the paste (formulation 1LE) and the fine suspension (formulation 2LE) during storage.

3 Stabilities of formulations 1LE and 2LE

The stabilities of the lutein esters were determined for formulations 1LE (as paste with polyoxyethylene sorbitan monooleate) and 2LE (as finely dispersed suspension with polyoxyethylene sorbitan monooleate and medium-chain triglyceride oil) in water, for the possibility of their incorporation into beverages or other aqueous media. Formulations 1LE and 2LE with 20 mg/L lutein esters were dissolved in ultra-pure water, with their stabilities monitored over 80 days. The solubilities were determined as the concentration of the active compound that was distributed into the aqueous phase and cannot be removed therein from sedimentation, which is a relevant parameter for the delivery of lutein in liquid products.

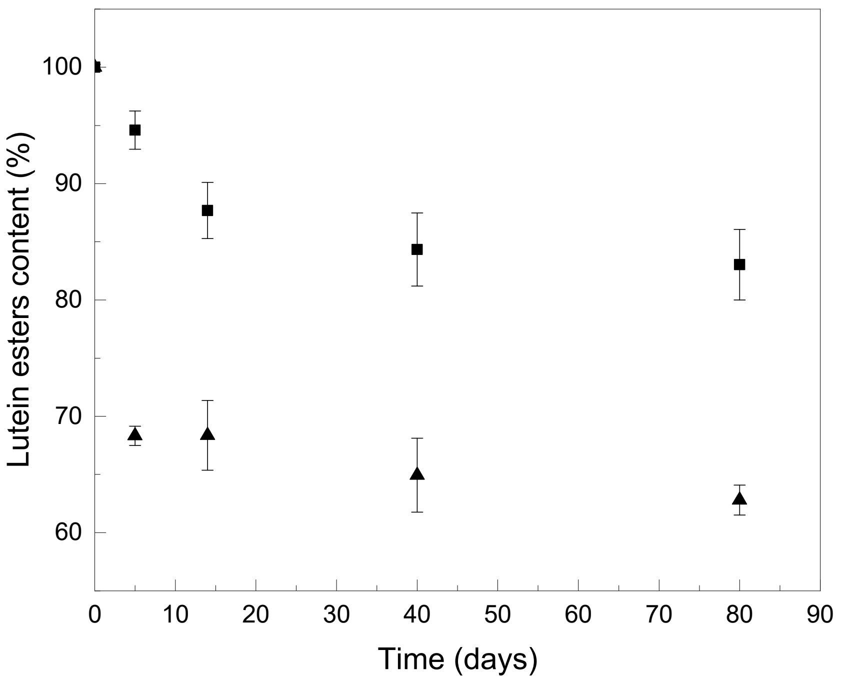

Figure S5: Stabilities of 20 mg/L lutein esters in formulations 1LE (▲) and 2LE (■) in water when stored at room temperature in the dark. Data are means ±standard deviation (n = 3). The lutein esters concentrations were determined from absorbance at 446 nm. The lutein esters were more stable in formulation 2LE than in formulation 1LE. The water dispersion of formulation 2LE contained 85% of the initial lutein esters concentration after 80 days, while that of formulation 1LE contained only 65% after the same time period. Formulation 2LE was expected to be more stable in water than formulation 1LE as it was already more stable in the prepared form. It appears that addition of the medium-chain triglyceride oil provided greater protection due to the entrapment within micelle-like units.

4 Solubility

The solubilities were determined as the amounts of stably dispersed active component (i.e., lutein) in solution for the formulations with polyoxyethylene sorbitan monooleate, thus indicating the amount of lutein that can be delivered within the liquid phase. Here, 100 mg formulations 1LE and 2LE were dissolved in 10 mL filtered ultrapure water. After 5 min centrifugation at 2590× *g* the supernatants were dried in 10 mL beakers at 105 °C to constant mass.

The solubility of non-encapsulated lutein in distilled water has been reported to be 0.197 mg/L at 37 °C (Zhao et al., 2018). These solubility tests showed improved solubility of encapsulated lutein in terms of its increased dispersion into the water phase. The solubility of lutein at room temperature (25 °C) as formulation 1LE was 20 mg/L, and for formulation 2LE, 120 mg/L. Addition of the medium-chain triglyceride oil improved the lutein solubility, as it was entrapped in this finely dispersed formulation. The lutein was about six-fold more soluble as formulation 2LE than as formulation 1LE. However, this lower solubility of lutein for formulation 1LE at room temperature was also 100-fold more soluble than that for the actual solubilisation of free lutein at 37 °C.

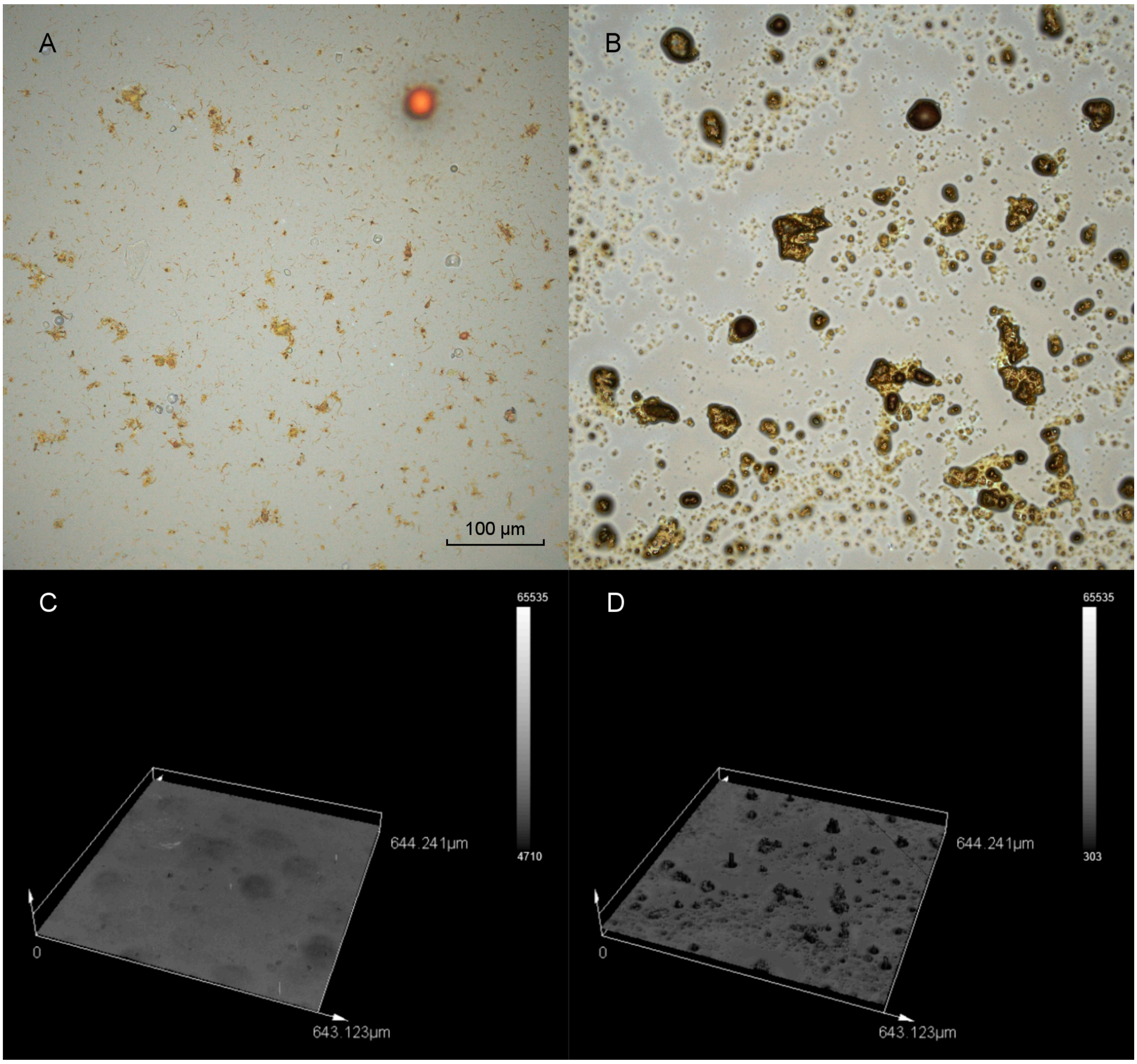

Figure S6: Micrographs of samples 1LE (A and C) and 2LE (B and D) via light microscopy at 228 × magnification (A and B) and corresponding 3D laser scans at 452 × magnification (C and D).
